# Weak preservation of local neutral substitution rates across mammalian genomes

**DOI:** 10.1186/1471-2148-9-89

**Published:** 2009-05-05

**Authors:** Hideo Imamura, John E Karro, Jeffrey H Chuang

**Affiliations:** 1 Boston College, Department of Biology, Chestnut Hill, MA, 02467 USA; 2 Instituut voor Tropische Geneeskunde, B-2000 Antwerpen, Belgium; 3 The Wellcome Trust Sanger Institute, Hinxton, Cambridge, CB10 1SA, UK; 4 Miami University of Ohio, Department of Computer Science & Systems Analysis and Department of Microbiology, Oxford, OH, 45056 USA

## Abstract

**Background:**

The rate at which neutral (non-functional) bases undergo substitution is highly dependent on their location within a genome. However, it is not clear how fast these location-dependent rates change, or to what extent the substitution rate *patterns *are conserved between lineages. To address this question, which is critical not only for understanding the substitution process but also for evaluating phylogenetic footprinting algorithms, we examine ancestral repeats: a predominantly neutral dataset with a significantly higher genomic density than other datasets commonly used to study substitution rate variation. Using this repeat data, we measure the extent to which orthologous ancestral repeat sequences exhibit similar substitution patterns in separate mammalian lineages, allowing us to ascertain how well local substitution rates have been preserved across species.

**Results:**

We calculated substitution rates for each ancestral repeat in each of three independent mammalian lineages (primate – from human/macaque alignments, rodent – from mouse/rat alignments, and laurasiatheria – from dog/cow alignments). We then measured the correlation of local substitution rates among these lineages. Overall we found the correlations between lineages to be statistically significant, but too weak to have much predictive power (*r*^2 ^<*5%*). These correlations were found to be primarily driven by regional effects at the scale of several hundred kb or larger. A few repeat classes (e.g. 7SK, Charlie8, and MER121) also exhibited stronger conservation of rate patterns, likely due to the effect of repeat-specific purifying selection. These classes should be excluded when estimating local neutral substitution rates.

**Conclusion:**

Although local neutral substitution rates have some correlations among mammalian species, these correlations have little predictive power on the scale of individual repeats. This indicates that local substitution rates have changed significantly among the lineages we have studied, and are likely to have changed even more for more diverged lineages. The correlations that do persist are too weak to be responsible for many of the highly conserved elements found by phylogenetic footprinting algorithms, leading us to conclude that such elements must be conserved due to selective forces.

## Background

Understanding neutral substitution rates is of fundamental importance in understanding the evolutionary process, as these rates define how the individual nucleotides and organization of genomes change [1,2]. Neutral substitution rates also have an important practical relevance for functional genomics, since they provide a threshold for inferring selective pressure from cross-species sequence conservation [3]. But because these rates vary by location along mammalian genomes, estimation of local rates is difficult and has been the subject of much study [4-7].

One approach towards a better understanding of neutral substitution rates is to measure how well these local rates are conserved across species [1,8-14]. A lack of rate conservation would imply that rates are dominated by lineage-specific (short-term) behavior. On the other hand, the presence of rate conservation would imply that substitution rates are determined by shared local features that might be retained in the orthologous loci of each species (e.g. base composition, recombination rate, pattern of nearby gene expression, etc.; see review by [15]). Furthermore, if local neutral substitution rates in one species were similar to the orthologous rates in another, this could result in cross-species sequence conservation at a locus resulting from neutral effects. Accounting for such effects would be important in evaluating phylogenetic footprinting calculations, which use sequence conservation to identify functional sequences[16].

Previous studies of neutral substitution rate conservation have focused on the rate of change of synonymous coding sites – presumed to undergo substitution at approximately the local neutral rate [1,9-13,17]. Synonymous substitution rates have been found to be correlated among several mammalian species, including mouse, rat, human, and dog. However, these correlations stem largely from genes associated with gene regulation, and much of the previously observed correlations are likely due to selection on the synonymous sites of such genes [1]. Because some synonymous sites are known to be under selection [18], as evidenced by studies of mRNA structure, splice sites, transcription level, and silent substitution fixation probabilities, decomposing the selective and neutral aspects of such sites can be difficult. These considerations, taken with the low density of such sites in the genome (0.4% of the human genome, separated by intergenic distances of 100–200 kb), suggest the value in measuring the neutral substitution rate through the use of other datasets.

An alternative source of data is provided by ancestral repeats (ARs), the dead remnants of transposable elements [2-5,7,19,20]. ARs are typically nonfunctional and occupy more than 40% of many mammalian genomes. This dense genomic coverage allows one to discern finer details of the neutral rate structure than is possible through the use of sparsely distributed synonymous sites. However, the use of ARs has some caveats. Fast evolving repeats may be too diverged to be recognized as ancient repeat elements. Repeats older than approximately 200 Myr cannot be identified [21], and younger repeats may also be missed in lineages that have a higher overall substitution rate (e.g. rodent [22]). RepeatMasker, the standard program for identifying repeats [23], fails to identify degenerated elements having more than 30–35% mismatches at typical repeat lengths [22]. And while ARs are frequently non-functional, they are not *always *non-functional [24]. Such effects have the potential to bias conclusions built on AR-based substitution rate calculations, thus must be understood and accounted for.

### Outline of the paper

In this work, we analyze the correlations of local neutral substitution rates in three independent mammalian lineages using a stringent set of ancestral repeat sequences obtained from UCSC mammalian alignments. We first examine the correlation of substitution rates from all orthologous repeats. From this we find that, although local neutral substitution rates exhibit statistically significant correlations across these lineages, these effects are too weak to have much practical predictive power (r^2 ^< 5%). We then resolve contributions from different length scales in the genome – determining whether the correlations are due to broad regional similarities, or just the singular behavior of individual repeat elements. We find that there are substantial regional effects on the scale of 10–500 kb, as well as additional effects at shorter length scales. Following this, we investigate the behavior of individual AR families, finding that although most families exhibit weak correlations, a few appear to be more strongly correlated due to the influence of purifying selection. Finally, we demonstrate the robustness of our observations to variations in methodology, such as CpG corrections, different substitution rate models, and dataset choice.

## Results

Here we briefly summarize our Methods. Our calculations are based on the alignment of ARs in the 17-way multiple alignment blocks from the UCSC Genome database [25]. We calculated the rate for a particular lineage from the alignments of pairs of species within that lineage (specifically: human against macaque for the primate lineage, mouse against rat for the rodent lineage, and dog against cow for the laurasiatheria lineage). We used only repeats that have been annotated by RepeatMasker in both of the species relevant to each calculation [6] – a more stringent filtering method than other approaches. Simple and low complexity repeats were excluded. To correct for uncertainty in the multiple alignment, we discarded blocks whose synteny was inconsistent with local orthologous genes. We restricted our analysis to autosomes in order to avoid the biases potentially introduced by sex chromosome recombination rate [26]. Using these aligned repeat elements, we measured the raw fraction of differing sites and normalized these rates with a finite size correction, yielding a z-score substitution rate for each repeat in each lineage [1]. Hence each z-score rate quantifies the deviation of the given repeat's substitution rate from the genomic mean.

### Rate Correlations Across Species

We first measured substitution rates in ancestral repeats from three lineages on a whole-genome scale. The resulting dataset has: ~740,000 repeats from the human/macaque alignment, covering 92.8 Mb; ~120,000 repeats from the mouse/rat alignment, covering 12.6 Mb; and ~620,000 repeats from the dog/cow alignment covering 63 Mb. Calculating raw substitution rates (fraction of differing sites), we find rates of 0.0570, 0.1277 and 0.2416 for human/macaque, mouse/rat and dog/cow respectively (Table 1).

**Table 1:** Genome-wide raw substitution rates (fraction of differing sites) and the ancestral repeat dataset size for each lineage, with and without CpG sites.

	**All bases**		**CpG removed**	
	Sub. rate	Size (bp)	Sub. Rate	Size (bp)
Primate (Human/Macaque)	0.0570	92.8 M	0.0494	90.9 M
Rodant (Rat/Mouse)	0.1277	12.6 M	0.1170	12.3 M
Laurasiatheria (Cow/Dog)	0.2416	63.0 M	0.2326	61.8 M

**Figure 1:**
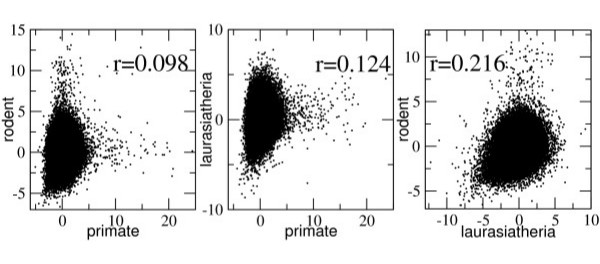
**Comparisons of substitution rates in orthologous ancestral repeat sequences from separate mammalian lineages**. Z-score normalized rates for each repeat were calculated in the three lineages: primate, rodent and laurasiatheria. In each pair-wise comparison of lineages (the three graphs), rates are shown for every ancestral repeat having well-defined orthology among the four species. Pearson correlations r for each pair of lineages were found to be significant but with low predictive power (r^2 ^< 5%) in each of the three comparisons.

We then measured the correlation of ancestral repeat substitution rates for orthologous loci in the three lineages. Overall, the Pearson correlations of orthologous local substitution rates (z-score normalized) are small for all three lineage comparisons: (*r *= 0.098 for primate vs. rodent, 0.124 for primate vs. laurasiatheria, and 0.216 for laurasiatheria vs. rodent), though they are significantly different from zero (details are given in Additional file 1). In Figure 1 we show scatter plots of the z-score rates in pairs of lineages. None of the plots show any strong linear trend, visually supporting the low correlation values. These correlations are quite similar on each chromosome as well (data not shown).

These small correlation coefficients are robust to the substitution rate model – other rate inference models yield correlation coefficients which are either comparable in value or weaker. Using the raw substitution rate ***μ***, we found slightly weaker correlations: primate – rodent 0.092, primate – laurasiatheria 0.083, laurasiatheria – rodent 0.155. Also, application of the Jukes-Cantor model to correct for the effect of multiple substitutions per site resulted in even weaker correlations (primate – rodent: r = 0.058; primate – laurasiatheria: r = 0.083; laurasiatheria – rodent; r = 0.135).

Although these correlations have statistically significant p-values, they have relatively little predictive power. The *r*^2 ^values, which indicate the fraction of the variance in the rates in one lineage explained by the rates in another lineage, are extremely weak: primate – rodent 0.96%, primate – laurasiatheria 1.24%, and laurasiatheria – rodent 4.6% for z-score rates, and even less for other rate measures. For raw substitution rates, the standard deviations of rates are each only a fraction of the genome-wide mean values (40% of the mean for primate, 32% for rodent, and 21% for laurasiatheria). Consequently, the low *r*^2 ^values indicate that even a repeat with a substitution rate that is multiple standard deviations away from the mean in one lineage would not be expected to have a raw substitution rate much different from the mean in another lineage.

### The Scale of Correlation

While we have observed correlations between local rates, it is not yet clear whether this is due to broad regional effects or to outlying rate values of just a few repeats. Substitution rates in any given genome are known to vary by region (in blocks as large as 10 Mb [17,27]), and if this regional structure is consistent across genomes it could explain the observed correlations. However, if the regional structure is not consistent across genomes, correlations might instead be explained by a few intermittent repeats that are unusually correlated due to selective pressures.

To determine the importance of regional effects on the correlations, we employed a procedure that corrects for potential regional effects. In the original method, we normalized the substitution rate of each repeat with respect to the genome wide average substitution rate. In the modified procedure, we instead normalized the substitution rate of each repeat with respect to the average substitution rate of all repeats within a local bin (see Methods for details). We used 12 different bin sizes, ranging from 25 Mb down to 2.5 kb. Our expectation was that for large bin sizes, correlations would behave in a manner similar to the original genome-wide correlations. As bin sizes decreased, we expected that local regional effects would be subsumed by the local normalization, causing correlations to decrease.

**Figure 2:**
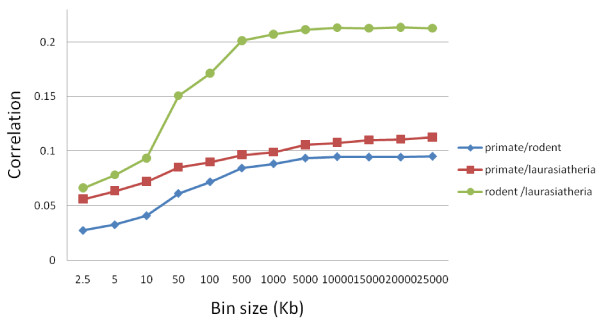
**Scaling behavior of rate correlations**. To determine the importance of regional effects on the correlations, we normalized the substitution rate of each repeat with respect to the average substitution rate in its local neighborhood, varying the bin size and hence the scope of the neighborhood. We then recalculated the correlations as a function of bin size. While the correlations are stable for bin sizes larger than 500 kb, they decrease in all three comparisons as the bin size becomes smaller. These results indicate that much of the correlations are due to regional effects on the scale of ~10–500 kb.

The results of this procedure are shown in Figure 2. Indeed, we found that the correlation decreased as the size of the local normalizing bin decreased. For bin sizes of 1 Mb–5 Mb, the correlations have a value about 90% of the genome-wide correlations. At ~10–50 kb, the correlations are one half of their full correlation values. Correlations decrease monotonically with decreasing bin size.

These results, together with Figure 2, indicate that much of the correlation is due to regional effects on the order of ~10–500 kb, thus acting on a more limited scale than the factors dictating substitution rates (there are blocks of up to ~10 Mb in which the observed substitution rate is relatively consistent[17]). Why do these scales differ? It appears that lineage-specific evolution has altered the regional pattern of substitution over time, causing the longest blocks to differ from one species to the next.

### Repeat Classes and Selection

**Figure 3:**
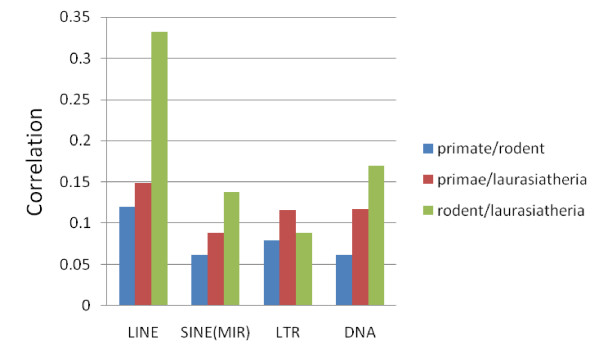
**Rate correlations in four major repeat classes**. LINEs have slightly higher correlations than other repeat classes, likely because a number of them are under selective pressures.

We next examined the substitution rate correlation in specific repeat classes and subclasses (Figure 3). We noticed that of the four major repeat classes (LINEs, SINEs, LTRs and DNA), LINEs have a slightly higher correlation than do the other classes. This effect is particularly apparent in the rodent – laurasiatheria comparison. Nevertheless, the *r*^2 ^value for LINEs in this comparison is still less than 11%; the actual substitution rates of LINEs do not differ noticeably from the other classes. (For details see Additional file 2.)

Subclasses within each of these main classes show substantial diversity in their correlations. Most subclasses exhibit correlations smaller than the genome wide correlation (Additional File 3). Thus low *r*^2 ^values are the norm, in spite of the varying base compositions and dinucleotide contents in each repeat subclass. However, a few have higher correlations. For example, among the 20 most abundant repeat subclasses (Additional File 3), those with above average correlations are MIR3, L3, L4, L1M5, and L1MEc in primate – rodent; L3 and L1ME4a in primate – laurasiatheria; and L3, L2, L4 and MIR3 in rodent – laurasiatheria. So multiple LINE subclasses contribute to the strong overall correlation for LINEs in Figure 3.

**Figure 4:**
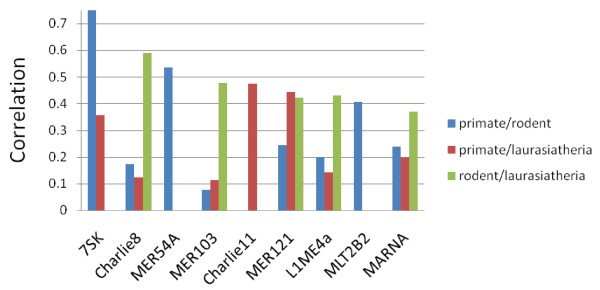
**Repeat subclasses with high rate correlations**. The repeats shown are those having correlation of ≥ 0.35 in at least one of the three lineage comparisons. Several of these (7SK, Charlie8, MER103, Charlie11, MER121, and MARNA), are likely to have been influenced by purifying selection, as they also exhibit lower than average substitution rates in all three lineages.

Some less abundant repeat subclasses also have strong rate correlations, and a number of subclasses with strong correlations appear to have been influenced by natural selection. For example, the most correlated repeat subclasses for primate – laurasiatheria, regardless of abundance, are listed in Table 2 (with data for the other two comparisons given in Additional file 4). These have been sorted by z-score, where a positive z-score indicates that the subclass has a stronger correlation than the genome-wide correlation (see Methods). In addition, repeat subclasses with the very strongest correlations (r > 0.35 in at least one of the three lineage comparisons) are shown in Figure 4. Notably, the repeat subclasses 7SK, Charlie8, MER103, Charlie11, MER121, and MARNA in Figure 4, as well as L3b from Table 2, all exhibit lower than average substitution rates in all three lineages (Additional File 5). This strongly suggests the influence of selective pressure on these families. Several of these repeat subclasses have been previously suggested to be functional as well [28-30]. The highest correlation for any repeat subclass is for the RNA repeat subclass 7SK, in the primate – rodent comparison. 7SK is known to mediate the inhibition of general transcription elongation factor P-TEFb by the HEXIM1 protein [31].

**Table 2:** Repeat subclasses with the top 10 strongest rate correlations (sorted by z-score) and with p-value < 0.001.

Subclass	Class	Blocks	Corr.	p-value	z-score
Charlie11	DNA/MER1_type	96	0.475	9.80E-07	3.278
MER121	Unknown	865	0.454	2.65E-45	2.890
7SK	RNA	98	0.357	0.0003	2.170
L3b	LINE/CR1	2394	0.282	3.04E-45	1.466
MER106B	DNA/MER1_type	313	0.282	3.90E-07	1.460
L1MB5	LINE/L1	2506	0.240	2.31E-34	1.071
LTR16B	LTR/ERVL	464	0.217	2.26E-06	0.853
L1MC1	LINE/L1	1260	0.208	8.22E-14	0.766
MARNA	DNA/Mariner	1595	0.204	1.69E-16	0.729
MER90a	LTR/ERV1	553	0.198	2.44E-06	0.678

Here we show the results comparing primate and laurasiatheria substitution rates; more extensive data are available in Additional File 4.

### Robustness of Correlations

#### Robustness to CpG removal

It is well-known that CpG dinucleotides are subjected to higher substitution rates because of the hypermutability of methlated cytosine in a CpG dinucleotide [32]. To account for this effect, we masked out the CpG dinucleotides in our alignments and recalculated the rates. As expected, the overall raw substitution rates after masking were lower (0.0494, 0.117 and 0.232 for primate, rodent and laurasiatheria, respectively – Table 1). The correlations of the substitution rates were, however, not noticeably affected. The r^2 ^values remained < 5% in all cases (primate – rodent: r = 0.103, primate – laurasiatheria: r = 0.105, laurasiatheria – rodent: r = 0.223. Details are in Additional file 6).

#### Robustness to orthology constraint

Our method of defining the dataset for each lineage comparison (i.e. using those aligned repeats with identical RepeatMasker annotation in the four species in question) is subject to a sampling bias that depends on species divergence. For more diverged species pairs (e.g. cow-dog) the orthologous sequences will differ more and alignable sequences may tend to be more conserved [33]. This could introduce a slight distortion into the dataset, which would explain the stronger correlations for comparisons involving cow-dog.

To test the strength of this effect, we modified our procedure to treat all lineages equally. In the altered procedure, we identified all orthologous repeats with consistent RepeatMasker annotations in all six species and then measured correlations in pairs of lineages using only data from this set. This resulted in correlation values of primate – rodent: r = 0.111, primate – laurasiatheria: r = 0.190, and laurasiatheria – rodent: r = 0.212. These correlations are only slightly stronger than those for the original calculations, and *r*^2 ^remains less than 4.5% in all cases. This indicates that sampling bias is not a serious complication.

#### Robustness to reference genome

When computing substitution rates for a particular lineage comparison, we implicitly required that the ARs be orthologous in all four species *as well as human *(if human is not one of the four). This additional requirement is an implicit effect of using the UCSC MultiZ alignments, which uses the human genome as a reference, but has the potential to introduce a bias into our results.

To test the impact of using human as the reference genome on the correlations, we recalculated them from a 17-way multiple alignment with mouse as the reference (one of the alternative MultiZ alignments available from UCSC). Once again, all *r*^2 ^values were small (< 0.06). The observed Pearson correlations were primate – rodent: 0.111, primate – laurasiatheria: 0.166, and laurasiatheria – rodent: 0.231, which are similar to the results when using human as the reference. Repeat subclasses that showed strong correlation when calculated from the human reference also did so when using the mouse reference – despite the considerably smaller dataset resulting from use of the mouse reference. We conclude that our results do not significantly depend on which reference genome is used for the alignments.

## Discussion

### Weak correlations

Overall we have found that correlation effects are weak (r^2 ^≲ 5%), implying that knowledge of the local substitution rate in one lineage has little impact on predicting substitution rates in another lineage. This result is robust in comparisons involving primate, rodent, and laurasiatheria, and it is not sensitive to CpG effects, orthology constraints, or the choice of reference genome. How general is this conclusion throughout the mammals? We chose the six species in this paper because they are in independent lineages at varying phylogenetic distances, are among the best sequenced genomes, and have high fractions of aligned sequence that includes several thousand repeats per chromosome even in the genomes with the least data available. These characteristics suggest that our findings are representative of the mammalian phylogeny. However, it is possible the species we have analyzed contain some lineage-specific behaviors. For example, it is known that the rodent lineage has more lineage-specific repeats and fewer ancestral repeats than other mammals, as well as a higher overall substitution rate [22]. The relatively low divergence between human and macaque could also be a concern, though this is in principle accounted for by the z-score rate normalizations.

To further test the robustness of the results, we analyzed the correlation of ancestral repeat substitution rates in the laurasiatheria lineage vs. the independent afrotheria lineage (represented by tenrec and elephant). This analysis was motivated by a recent work of Prakash and Tompa [34], who reported that the tenrec/elephant tracks of the UCSC 17-way alignments are comparable in quality to dog/cow, and in coverage to mouse/rat. The laurasiatheria normalized rates correlate with the afrotheria rates at r = 0.27 (r^2 ^= 7.3%), which is only modestly larger than the largest correlation we had previously observed. This supports the general conclusion of weak correlations. For the variety of evolutionary distances between various mammalian lineages[17,35], our intuitive expectation is that lineages separated by greater distances should have weaker correlations.

Are substitution rates correlated in species that are more closely related than the lineages we have examined here? Orthologous human and chimp repeats are so closely related that stochastic effects can make it difficult to infer the local substitution rate (the raw 4-fold site substitution rate is ~5× larger in human-macaque than in human-chimpanzee) [12]. However, Hodgkinson et al. recently found orthologous repeats exhibiting similar SNP patterns in human and chimp, suggesting that, at least for some loci, substitution rates can be preserved for a few million years[36].

One potential concern about the use of repetitive sequences is that their substitution rates may have been more influenced by gene conversion than other neutral segments. Our data show that virtually all repeat families, regardless of their susceptibility to gene conversion, exhibit weak rate correlations. If gene conversion were relevant to our findings, we would expect the magnitude of its influence on a particular family to be proportional to the copy number of that family. However, for the top 20 most abundant repeat families, every family has a primate-rodent correlation < 0.16, despite a 25-fold range in copy numbers (details and other lineage comparisons in Additional File 3). Gene conversion is also known to engender higher substitution rates, as well as increased GC content resulting from biased conversion[37]. Though among the 20 most abundant families, rate correlations show little sign of association with either GC content (primate-rodent association p-value = 0.6) or with substitution rate (p-value = 0.2).

Our results shed light on previous observations of the correlations of synonymous substitution rate between lineages[1]. Synonymous substitution rates are more difficult to analyze than substitution rates in repeats, as synonymous rates are noticeably influenced by sites subject to selection. While synonymous rate correlations are larger than for repeats (r^2^~9% between the human-dog lineage and the rat-mouse lineage), the correlations are still relatively small. This suggests that our observations for repeats are representative of how neutral substitution rates change over time, rather than some peculiarity of repeat sequences.

### Relevance for Phylogenetic Footprinting

While the correlations we observe both here and in previous analysis of synonymous substitution rates [1] are all statistically significant, the correlations are too weak to provide much predictive power between lineages. This low predictive power is of crucial importance in the context of phylogenetic footprinting – the detection of functional sequences based on their conservation beyond neutral expectations [38-40]. The implicit assumption in phylogenetic footprinting is that sequences detected by the method have strong conservation because they are under natural selection.

Theoretically, if local neutral rates are correlated across species, a conserved block could arise from the persistence of orthologous neutral substitution cold spots without involving selection. However, the low *r*^2 ^values we report here show that persistent substitution cold spots are unlikely to be responsible for more than a small fraction of sequences which have been identified by phylogenetic footprinting. Furthermore, we have found that much of the preserved rate structure occurs at scales of several hundred kb or longer (a related study by Tykucheva et al. also found rate correlations across species in a study of 1 Mb blocks using a different methodology and dataset [3]). This is a scale much larger than the elements identified by phylogenetic footprinting, which are typically only a few hundred bp long. This discordance in length scales further limits persistent substitution coldspots as an explanation for elements detected by phylogenetic footprinting.

### Selective Pressure

Although our intent in analyzing this ancestral repeat data was to study neutral effects, selection still appears to be a factor. The repeats with the lowest substitution rates – those most likely to be under negative selective pressure – contribute more strongly to the correlations than other repeats. On the other hand, lineage specific selection could also cause divergent substitution rates in some repeats. But given the neutrality of a majority of repeats[2-4,19], this should on the whole be a minor effect.

Overall, repeats with strong phastCons scores – a measure of selective pressure via phylogenetic footprinting [40] – have correlations substantially greater than those of the remaining repeats. For repeats with phastCons score > 0.9 (~10% of our data) we see *r *= *0.113 *for primate – rodent, *0.173 *for primate – laurasiatheria, and *0.247 *for laurasiatheria – rodent. For the remaining repeats, we have *r *= *0.045, 0.090 *and *0.064 *respectively (details in Additional file 7). Such results confirm that purifying selection is responsible for much of the substitution rate correlations. We find similar results when we compare correlations in the repeats with the top 10% vs those with the lowest 10% of substitution rates. These results imply that the r^2 ^≲ 7% correlations are an *upper bound *on the contributions from persistent neutral substitution rates. The true neutral component should be even weaker.

The influence of selection is consistent with recent studies that have found evidence of the functionality of certain repeats or repeat classes. For example, Siepel et al. have estimated that about 3.7% of all elements with high phastCons scores are ancestral repeats [40]. Some of these functions may be cis-regulatory [28] or the result of regaining of transposon activity, known as domestication [24,41-46]. It has been estimated that about 0.05 of human transposons are currently active [47].

A number of repeat classes with strong substitution rate correlations are likely to be under selection. For example, Kamal et al. reported that MER121, L3b, L3, MARNA, MER103, MER102b, Charlie8, L1ME4a are highly conserved in human, dog, rat, and mouse [28]. These groups have some of the highest correlations in our data set. Lowe et al. found functional elements with strong sequence conservation derived from repeats close to developmental genes, notably MER121, L3b and L3 (CR1), L4(RTE) and MARNA(Mariner)[29], which also exhibit strong correlations in our data. Additionally, we found a few repeat subclasses with high rate correlation that have not been previously discussed; these include 7Sk, MER54A, Charlie11 and MLT2B2. It would be worthwhile to further investigate their possible functionality. Jurka examined the overlap of the repetitive families with the evolutionarily conserved, potential *cis*-regulatory regions. Unfortunately, with the exception of MER1232, repeat elements discussed in his paper are not significantly present in our data [30].

What do these repeats elements under strong selection tell us about phylogenetic footprinting? These elements can artificially suppress the "neutral" substitution rate if there are a large number of them in a given block. In such a case, removing such repeats will improve the estimation of the background model used in the phylogenetic footprinting.

## Conclusion

In this work we have shown that mammalian local neutral substitution rates are largely lineage-specific, suggesting that it is best to estimate substitution rates from single-species data whenever possible [2,5,7,19]. Some correlations in substitution rates in repeat sequences exist, but, as for silent sites, these have been influenced by nucleotides under purifying selection. Much of the remaining neutral correlations are due to effects at large length scales from 10–500 kb. These findings indicate that most highly conserved mammalian sequences detected are indeed under natural selection, rather than the result of persistent local neutral substitution rates.

## Methods

### Dataset

We obtained multiple alignments of 17 vertebrates, calculated both from a human reference and a mouse reference, from the UCSC genome browser (hg18/multiz17way and mm8/multiz17way). From that data we extracted the alignments of human, macaque, mouse, rat, cow and dog. Additionally we obtained the RepeatMasker repeat annotations for each of these genomes from the UCSC genome browser. These annotations were generated using the "sensitive" option. We excluded simple and low complexity repeats, satellites, and primate specific Alu repeat families. We used Perl scripts and Berkeley DB to store and process repeat annotation data in each species.

To improve the quality of our dataset, we restricted our analysis to repeats having synteny consistent with nearby genes. We constructed a synteny map from one-to-one orthologous genes between both human/dog and human/mouse, which we downloaded from Biomart [48] in Ensembl [49]. 0.5% of total alignment blocks were discarded because they were non-syntenic. We also used AutoGRAPH [50], a web based synteny visualization tool, to inspect synteny structures.

When comparing two lineages we chose members of our repeat dataset using the following conditions: 1) Each repeat has an orthologous sequence in all four of the species in the two lineages, 2) Each repeat has an orthologous sequence in the reference sequence (human or mouse, depending on the run), and 3) Each repeat has an identical RepeatMasker classification in the reference sequence and all four alignment species. The first condition influences the background substitution rate, since the average rate obtained from 2-way ortholog alignments is typically higher than those of 4-way ortholog alignments. The second condition allows us to derive a consistent syntenic set in each comparison of lineages. The third condition provides stringency in the dataset, a feature which distinguishes our study from previous investigations [3]. The stringency of our conditions may bias the data set toward conserved repeat elements. However, without stringency we risk being adversely influenced by the large fraction of repeats with questionable orthology (~60% of human repeats are aligned to non-repeats in mouse and rat in the UCSC alignments). Our conditions are overall similar to those of prior studies of orthologous repeat elements [28,33,51,52].

### Normalized substitution rates

We characterized local substitution rates and measured cross species correlations using a z-score model which has been described previously [1], for several reasons. The z-score approach has the advantage of correcting for finite size effects in each block. To calculate the z-score, we first measured the fraction of non-matching sites (the raw substitution rate) for a given aligned orthologous pair. We then normalized based on the expected standard deviation given the repeat length. The expected standard deviation of a repeat containing N bases was calculated from that expected of a binomial distribution,  where the bar indicates a genome wide average. This standard deviation was then used to rescale the raw substitution rate ***μ*_*i *_**to a normalized rate  for a locus i, where the bar indicates a genome wide average. This yields a rate distribution that is zero-centered and whose standard deviation is of order 1. This zero-centering makes the rates insensitive to features that influence absolute substitution rates, such as the generation times in each lineage[53].

### The scale of correlation

We first calculated the average raw substitution rate in bins (of variable size s) along the genome. We then used these local average substitution rates to calculate normalized rates ***ρ***, as an alternative to the rates calculated using the genome-wide rate  for the normalization. Correlations of ***ρ ***across lineages were then calculated as a function of the bin size s. Let the raw substitution rate at repeat *i *be ***μ*_*i*_**, and let  indicate the average raw substitution rate of all repeats in a bin of size s around locus *i*. A binned normalized substitution rate can be then written as . By changing the bin size, we are able to measure how important different length scales are to the rate correlation.

### Correlation z-scores for repeat subclasses

The z-score for a repeat subclass is defined as *z *= (*r*_*sc *_- *r*)/*σ*(*r*_*sc*_) where *r*_*sc *_is the correlation of the repeat subclass (sc), *r *is the genome wide rate correlation, and *σ*(*r*_*sc*_) the standard deviation among the rate correlations over all repeat subclasses. This z-score is used to rank the strength of correlations among different classes. Positive z-scores indicate an above-average correlation and negative z-scores indicate below-average.

### PhastCons

PhastCons is a phylogenetic Hidden Markov Model which evaluates the strength of sequence conservation across species [40]. Vertebrate phastCons conservation scores for each base along the human genome were downloaded from the UCSC Genome Browser.

## Authors' contributions

HI participated in the design of the study, performed the data acquisition and analysis, and drafted the manuscript. JEK contributed to the analysis of the data and the writing of the manuscript. JHC conceived of the study, contributed to the analysis of the data, and contributed to the writing of the manuscript. All authors read and approved the final manuscript.

## Supplementary Material

Additional file 1 **Total base pairs, the number of blocks and the average size of blocks**. The table provides total base pairs, the number of blocks and the average size of blocks used for each lineage. Click here for file

Additional file 2 **Genome-wide average substitution rates and dataset sizes for four major repeat classes**. Description: The tables describe genome-wide average substitution rates and dataset sizes for LINE, SINE, LTR and DNA. Click here for file

Additional file 3 **The top 20 most abundant repeat subclasses**. The top 20 most abundant repeat subclasses are given for primate-rodent, primate-laurasiatheria, and laurasiatheria-rodent. Click here for file

Additional file 4 **Repeat subclasses of strong rate correlation among the other groups**. Two additional tables describe repeat subclasses of strong rate correlation between primate and rodent, and between rodent and laurasiatheria. Click here for file

Additional file 5 **Average substitution rates of the repeat subclasses 7SK, Charlie8, MER103, Charlie11, MER121, and MARNA**. The table shows lower than average substitution rates of the repeat subclasses in all three lineages. Click here for file

Additional file 6 **CpG removed data sets**. The file contains scattering plots and a table for total base pairs, the number of blocks and the average size of blocks used for each lineage. Click here for file

Additional file 7 **The analysis based on phastCons conservation score**. Statistics of correlations and raw data classified based on phastCons conservation score of 17 species alignments illustrate the effects of purifying selection. Click here for file
