# Supplementary material for: Weak preservation of local neutral substitution rates across mammalian genomes
Source: BMC Evol Biol. 2009 May 5;9:89. doi: 10.1186/1471-2148-9-89 (PMC2689173; doi:10.1186/1471-2148-9-89)
Supplement: Additional file 1 — Total base pairs, the number of blocks and the average size of blocks. The table provides total base pairs, the number of blocks and the average size of blocks used for each lineage. [file 1471-2148-9-89-S1.doc]

Additional file1

Total base pairs, the number of blocks and the average size of blocks.

Although the number of total bases in lineages is not the same, results do not noticeably depend on whether this is resolved by the use of trimmed blocks of the same size or untrimmed blocks of different sizes.

|  | primate | | rodent | primate | laurasia-theria | laurasiat-heria | rodent |
| --- | --- | --- | --- | --- | --- | --- | --- |
| Correlation | 0.098 | | | 0.124 | | 0.216 | |
| p-value | 1.4E-254 | | | 0 | | 0 | |
| Total block | 119009 | | | 646693 | | 49649 | |
| Total (bp) | 17.89M | 12.34M | | 77.13M | 63.04M | 6.17M | 5.22M |
| Average size (bp) | 150 | 103 | | 119 | 97 | 124 | 105 |
