# Supplementary material for: Weak preservation of local neutral substitution rates across mammalian genomes
Source: BMC Evol Biol. 2009 May 5;9:89. doi: 10.1186/1471-2148-9-89 (PMC2689173; doi:10.1186/1471-2148-9-89)
Supplement: Additional file 2 — Genome-wide average substitution rates and dataset sizes for four major repeat classes. Description: The tables describe genome-wide average substitution rates and dataset sizes for LINE, SINE, LTR and DNA. [file 1471-2148-9-89-S2.doc]

Additional file 2

Genome-wide average substitution rates and dataset sizes for four major repeat classes.

a)

|  | LINE | | MIR | | LTR | | DNA | |
| --- | --- | --- | --- | --- | --- | --- | --- | --- |
|  | Sub. rate | Size | Sub. rate | Size | Sub. rate | Size | Sub. rate | Size |
| Primate | 0.056 | 35.0Mbp | 0.054 | 21.0Mbp | 0.060 | 21.4Mbp | 0.057 | 15.2Mbp |
| Rodent | 0.127 | 5.5Mbp | 0.119 | 2.5Mbp | 0.136 | 2.9Mbp | 0.126 | 1.6Mbp |
| Laurasiatheria | 0.236 | 21.5Mbp | 0.235 | 15.3Mbp | 0.256 | 15.1Mbp | 0.241 | 11.4Mbp |

Total base pairs, the number of blocks and the average size of blocks for each class.

b)

| LINE | primate | rodent | primate | laurasia-theria | laurasia-theria | rodent |
| --- | --- | --- | --- | --- | --- | --- |
| Correlation | 0.120 | | 0.149 | | 0.332 | |
| p-value | 7.47e-167 | | 0.0 | | 0.0 | |
| Total block | 52217 | | 197317 | | 13638 | |
| Total (bp) | 8.43M | 5.46M | 26.94M | 21.50M | 1.49M | 1.21M |
| Average size (bp) | 175 | 113 | 145 | 115 | 153 | 124 |

c)

| SINE | primate | rodent | primate | laurasia-theria | laurasia-theria | rodent |
| --- | --- | --- | --- | --- | --- | --- |
| Correlation | 0.060 | | 0.085 | | 0.1388 | |
| p-value | 1.6e-28 | | 7.8e-315 | | 6.7e-75 | |
| Total block | 29741 | | 198195 | | 17225 | |
| Total (bp) | 3.08M | 2.33M | 18.15M | 15.06M | 1.62M | 1.46M |
| Average size (bp) | 103 | 78 | 91 | 76 | 94 | 85 |

d)

| LTR | primate | rodent | primate | laurasia-theria | laurasia-theria | rodent |
| --- | --- | --- | --- | --- | --- | --- |
| Correlation | 0.078969 | | 0.115971 | | 0.0880 | |
| p-value | 4.64e-36 | | 0.0 | | 1.56e-19 | |
| Total block | 25130 | | 137879 | | 10504 | |
| Total (bp) | 4.18M | 2.88M | 18.55M | 15.11M | 1.49M | 1.20M |
| Average size (bp) | 178 | 123 | 142 | 115 | 153 | 124 |

| DNA | primate | rodent | primate | laurasia-theria | laurasia-theria | rodent |
| --- | --- | --- | --- | --- | --- | --- |
| Correlation | 0.0612 | | 0.1165 | | 0.17002722 | |
| p-value | 2.54e-17 | | 0.0 | | 2.365e-72 | |
| Total block | 19069 | | 139970 | | 11034 | |
| Total (bp) | 2.16M | 1.63M | 13.45M | 11.33M | 1.11M | 0.97M |
| Average size (bp) | 122 | 92 | 101 | 85 | 109 | 95 |
