# Supplementary material for: Weak preservation of local neutral substitution rates across mammalian genomes
Source: BMC Evol Biol. 2009 May 5;9:89. doi: 10.1186/1471-2148-9-89 (PMC2689173; doi:10.1186/1471-2148-9-89)
Supplement: Additional file 3 — The top 20 most abundant repeat subclasses. The top 20 most abundant repeat subclasses are given for primate-rodent, primate-laurasiatheria, and laurasiatheria-rodent. [file 1471-2148-9-89-S3.doc]

Additional file 3

Among the top 20 most abundant repeat subclasses, only a few have larger than average correlations; that is, only a few subclasses have positive z-score for each quartet.

The z-score here is defined as
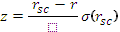
 where
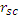
is a correlation of a repeat subclass(sc),
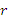
a genome wide rate correlation, and
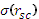
 the standard deviation among the rate correlation among all the repeat subclasses. This z-score is to qualitatively rank the strength of these correlations. The value of r is 0.098, 0.124, and 0.216 for the comparisons of primate-rodent, primate-laurasiatheria, and laurasiatheria-rodent, respectively.

a) Top 20 most abundant repeat subclasses between primate (P) and rodent (R).

| Class | Subclass | Ave. size | | Total  blocks | Total (bp) | | % | | Corr. (r) | p-value | z-score |
| --- | --- | --- | --- | --- | --- | --- | --- | --- | --- | --- | --- |
|  |  | P | R |  | P | R | P | R |  |  |  |
| LINE/L2 | L2 | 163 | 102 | 24062 | 3912021 | 2442230 | 0.2186 | 0.1979 | 0.0821 | 2.82E-37 | -0.298 |
| SINE/MIR | MIRb | 104 | 78 | 14316 | 1481318 | 1112448 | 0.0828 | 0.0901 | 0.0558 | 2.32E-11 | -0.790 |
| SINE/MIR | MIR | 107 | 81 | 12624 | 1349280 | 1020280 | 0.0754 | 0.0827 | 0.0357 | 5.97E-05 | -1.166 |
| DNA/MER1_type | MER5A | 93 | 72 | 3200 | 297637 | 229134 | 0.0166 | 0.0186 | 0.0418 | 0.018041 | -1.053 |
| SINE/MIR | MIR3 | 91 | 74 | 2650 | 241892 | 196495 | 0.0135 | 0.0159 | 0.1078 | 2.68E-08 | 0.183 |
| LINE/CR1 | L3 | 162 | 111 | 2616 | 424574 | 290868 | 0.0237 | 0.0236 | 0.1564 | 1.53E-15 | 1.094 |
| LTR/MaLR | MLT1D | 159 | 105 | 2570 | 409505 | 270904 | 0.0229 | 0.0219 | 0.0747 | 0.000131 | -0.436 |
| LTR/MaLR | MLT1A0 | 164 | 124 | 2538 | 417268 | 315606 | 0.0233 | 0.0256 | 0.0830 | 2.80E-05 | -0.280 |
| DNA/MER1_type | MER5B | 87 | 69 | 2105 | 182325 | 144187 | 0.0102 | 0.0117 | 0.0174 | 0.425983 | -1.510 |
| LTR/MaLR | MLT1C | 181 | 128 | 1964 | 355604 | 251771 | 0.0199 | 0.0204 | 0.0353 | 0.118022 | -1.175 |
| DNA/MER1_type | MER20 | 107 | 83 | 1805 | 192412 | 150238 | 0.0108 | 0.0122 | 0.0554 | 0.018653 | -0.798 |
| LTR/MaLR | MLT1B | 166 | 123 | 1731 | 286652 | 212777 | 0.0160 | 0.0172 | 0.0649 | 0.006908 | -0.620 |
| LINE/L1 | HAL1 | 214 | 123 | 1283 | 274496 | 158385 | 0.0153 | 0.0128 | 0.0532 | 0.056575 | -0.838 |
| LINE/RTE | L4 | 172 | 110 | 1268 | 218510 | 138788 | 0.0122 | 0.0112 | 0.1083 | 0.000111 | 0.194 |
| LINE/L1 | L1MC3 | 189 | 134 | 1240 | 234756 | 166414 | 0.0131 | 0.0135 | -0.0263 | 0.353999 | -2.329 |
| LINE/L1 | L1MC4 | 170 | 110 | 1157 | 196163 | 127570 | 0.0110 | 0.0103 | 0.0525 | 0.075401 | -0.853 |
| LINE/L1 | L1M5 | 179 | 117 | 1149 | 205719 | 134157 | 0.0115 | 0.0109 | 0.1059 | 0.000308 | 0.148 |
| DNA/MER1_type | MER58A | 112 | 93 | 1111 | 124313 | 102756 | 0.0069 | 0.0083 | -0.0647 | 0.031014 | -3.047 |
| LINE/L1 | L1ME1 | 178 | 127 | 1079 | 192111 | 137100 | 0.0107 | 0.0111 | 0.0649 | 0.033142 | -0.621 |
| LINE/L1 | L1MEc | 249 | 131 | 897 | 222962 | 117792 | 0.0125 | 0.0095 | 0.1569 | 1.09E-06 | 0.2444 |

b) Top 20 most abundant repeat subclasses between primate (P) and laurasiatheria (L).

| Class | Subclass | Ave. size | | Total  blocks | Total (bp) | | % | | Corr. (r) | p-value | z-score |
| --- | --- | --- | --- | --- | --- | --- | --- | --- | --- | --- | --- |
|  |  | P | L |  | P | L | P | L |  |  |  |
| SINE/MIR | MIRb | 92 | 76 | 95889 | 8811233 | 7245763 | 0.1142 | 0.1149 | 0.0822 | 3.12E-143 | -1.579 |
| SINE/MIR | MIR | 94 | 79 | 79032 | 7446656 | 6205664 | 0.0965 | 0.0984 | 0.0720 | 2.47E-91 | -1.962 |
| LINE/L2 | L2 | 152 | 117 | 27350 | 4163620 | 3186765 | 0.0540 | 0.0506 | 0.1240 | 3.74E-94 | 0.000 |
| SINE/MIR | MIR3 | 82 | 70 | 23274 | 1900091 | 1617436 | 0.0246 | 0.0257 | 0.1100 | 1.45E-63 | -0.528 |
| LINE/CR1 | L3 | 125 | 97 | 20963 | 2614606 | 2034025 | 0.0339 | 0.0323 | 0.1620 | 3.07E-123 | 1.434 |
| DNA/MER1_type | MER5A | 82 | 69 | 17518 | 1427016 | 1203978 | 0.0185 | 0.0191 | 0.0917 | 4.65E-34 | -1.217 |
| LTR/MaLR | MLT1D | 141 | 119 | 13951 | 1966044 | 1656479 | 0.0255 | 0.0263 | 0.0775 | 4.91E-20 | -1.755 |
| LINE/L1 | HAL1 | 164 | 122 | 13872 | 2277783 | 1689919 | 0.0295 | 0.0268 | 0.0822 | 3.20E-22 | -1.578 |
| DNA/MER1_type | MER5B | 77 | 65 | 12615 | 972737 | 816107 | 0.0126 | 0.0129 | 0.0584 | 5.35E-11 | -2.476 |
| LINE/L1 | L1MC4 | 144 | 113 | 11977 | 1727372 | 1357305 | 0.0224 | 0.0215 | 0.0781 | 1.10E-17 | -1.730 |
| DNA/MER1_type | MER20 | 99 | 84 | 11607 | 1146885 | 978930 | 0.0149 | 0.0155 | 0.0817 | 1.18E-18 | -1.596 |
| LINE/L1 | L1ME4a | 153 | 130 | 10882 | 1664045 | 1411624 | 0.0216 | 0.0224 | 0.1405 | 2.82E-48 | 0.621 |
| LTR/MaLR | MLT1C | 111 | 92 | 10700 | 1184196 | 982430 | 0.0154 | 0.0156 | 0.1172 | 1.28E-34 | -0.255 |
| LTR/MaLR | MLT1K | 134 | 104 | 9744 | 1304627 | 1008362 | 0.0169 | 0.0160 | 0.0949 | 6.24E-21 | -1.099 |
| LINE/L1 | L1MC5 | 126 | 101 | 9223 | 1159734 | 934670 | 0.0150 | 0.0148 | 0.0839 | 6.80E-16 | -1.512 |
| DNA/MER1_type | MER5A1 | 79 | 68 | 8909 | 702860 | 609035 | 0.0091 | 0.0097 | 0.0810 | 1.86E-14 | -1.621 |
| DNA/MER1_type | MER58A | 100 | 89 | 8399 | 843568 | 743925 | 0.0109 | 0.0118 | 0.0698 | 1.48E-10 | -2.044 |
| LINE/L1 | L1ME1 | 160 | 135 | 8184 | 1312364 | 1104862 | 0.0170 | 0.0175 | 0.1123 | 2.18E-24 | -0.441 |
| LTR/MaLR | MLT1J | 133 | 106 | 8154 | 1086489 | 860318 | 0.0141 | 0.0136 | 0.0635 | 9.57E-09 | -2.283 |
| LINE/L1 | L1M5 | 135 | 109 | 7893 | 1065876 | 857771 | 0.0138 | 0.0136 | 0.0998 | 3.78E-20 | -0.23699 |

c) Top 20 most abundant repeat subclasses between rodent (R) and laurasiatheria (L).

| Class | Subclass | Ave.size | | Total  blocks | Total (bp) | | % | | Corr. (r) | p-value | z-score |
| --- | --- | --- | --- | --- | --- | --- | --- | --- | --- | --- | --- |
|  |  | L | R |  | L | R | L | R |  |  |  |
| SINE/MIR | MIR | 94 | 84 | 7829 | 737025 | 661138 | 0.1194 | 0.1265 | 0.0932 | 2.16E-16 | -1.252 |
| SINE/MIR | MIRb | 97 | 87 | 7739 | 749045 | 675254 | 0.1214 | 0.1292 | 0.1150 | 1.76E-24 | -1.029 |
| DNA/MER1_type | MER5A | 85 | 75 | 2033 | 172581 | 153330 | 0.0280 | 0.0293 | 0.0948 | 1.87E-05 | -1.236 |
| LINE/CR1 | L3 | 144 | 117 | 1959 | 281082 | 228411 | 0.0455 | 0.0437 | 0.3249 | 2.23E-49 | 1.110 |
| LINE/L2 | L2 | 157 | 125 | 1914 | 300250 | 238556 | 0.0487 | 0.0456 | 0.2880 | 7.20E-38 | 0.734 |
| SINE/MIR | MIR3 | 86 | 81 | 1657 | 142288 | 133498 | 0.0231 | 0.0255 | 0.2532 | 1.19E-25 | 0.379 |
| LTR/MaLR | MLT1D | 143 | 114 | 1489 | 212645 | 169345 | 0.0345 | 0.0324 | 0.0451 | 0.081829 | -1.742 |
| DNA/MER1_type | MER5B | 80 | 74 | 1383 | 110202 | 101885 | 0.0179 | 0.0195 | 0.1196 | 8.26E-06 | -0.983 |
| DNA/MER1_type | MER20 | 98 | 89 | 1119 | 109123 | 99061 | 0.0177 | 0.0190 | 0.0259 | 0.387525 | -1.938 |
| LTR/MaLR | MLT1C | 164 | 134 | 1004 | 164599 | 134857 | 0.0267 | 0.0258 | 0.0266 | 0.399626 | -1.931 |
| LINE/RTE | L4 | 157 | 120 | 937 | 147390 | 112218 | 0.0239 | 0.0215 | 0.2713 | 2.82E-17 | 0.564 |
| LINE/L1 | HAL1 | 152 | 133 | 751 | 113889 | 99745 | 0.0185 | 0.0191 | 0.0545 | 0.137755 | -1.646 |
| LTR/MaLR | MLT1B | 175 | 131 | 742 | 129512 | 97060 | 0.0210 | 0.0186 | 0.0225 | 0.538712 | -1.973 |
| DNA/MER1_type | MER58A | 105 | 98 | 692 | 72420 | 67533 | 0.0117 | 0.0129 | 0.0795 | 0.036525 | -1.391 |
| LINE/L1 | L1MC4 | 154 | 124 | 615 | 94896 | 76511 | 0.0154 | 0.0146 | 0.0895 | 0.026496 | -1.290 |
| LINE/L1 | L1ME1 | 151 | 124 | 546 | 82352 | 67726 | 0.0133 | 0.0130 | 0.1410 | 0.000957 | -0.765 |
| LINE/L1 | L1MC3 | 150 | 134 | 486 | 73027 | 64924 | 0.0118 | 0.0124 | -0.0129 | 0.777294 | -2.333 |
| DNA/MER1_type | MER5A1 | 175 | 140 | 472 | 82750 | 66123 | 0.0134 | 0.0127 | 0.0197 | 0.681359 | -2.001 |
| LTR/MaLR | MLT1J | 149 | 115 | 463 | 68831 | 53272 | 0.0112 | 0.0102 | 0.0637 | 0.176143 | -1.552 |
| DNA/MER2_type | MER82 | 138 | 110 | 452 | 62254 | 49898 | 0.0101 | 0.0095 | 0.1426 | 1.77E-03 | -0.29347 |
