# Supplementary material for: Weak preservation of local neutral substitution rates across mammalian genomes
Source: BMC Evol Biol. 2009 May 5;9:89. doi: 10.1186/1471-2148-9-89 (PMC2689173; doi:10.1186/1471-2148-9-89)
Supplement: Additional file 4 — Repeat subclasses of strong rate correlation among the other groups. Two additional tables describe repeat subclasses of strong rate correlation between primate and rodent, and between rodent and laurasiatheria. [file 1471-2148-9-89-S4.doc]

Additional file 4

a)

| Subclass | Class | Blocks | Corr. | p-value | z-score |
| --- | --- | --- | --- | --- | --- |
| 7SK | RNA | 14 | 0.752 | 0.00118 | 2.49 |
| MER54A | LTR/ERVL | 49 | 0.531 | 9.32E-05 | 1.601 |
| MLT2B2 | LTR/ERVL | 151 | 0.406 | 2.17E-07 | 1.130 |
| MIRm | SINE/MIR | 151 | 0.321 | 5.65E-05 | 0.814 |
| LTR67 | LTR/ERVL | 109 | 0.310 | 0.000733 | 0.803 |
| MER171 | LTR/ERVL | 200 | 0.237 | 0.000712 | 0.502 |
| L3b | LINE/CR1 | 534 | 0.215 | 4.92E-07 | 0.421 |
| MARNA | DNA/Mariner | 143 | 0.204 | 0.014171 | 0.381 |
| MER121 | Unknown | 192 | 0.200 | 0.005306 | 0.363 |
|  |  |  |  |  |  |

Repeat subclasses of strong rate correlation between primate and rodent. P-values are those from standard Pearson correlation t-tests. Although MARNA has a slightly larger p-value, it is listed for comparison with other papers.

Criteria: z>0.3 or p-value better than 0.001.

b)

laurasiatheria- rodent

| Subclass | Class | Block | Corr. | p-value | z-score |
| --- | --- | --- | --- | --- | --- |
| Charlie8 | DNA/MER1_type | 52 | 0.588 | 4.5E-06 | 1.190 |
| MER103 | DNA | 57 | 0.412 | 1.4E-03 | 0.630 |
| L1ME4a | LINE/L1 | 364 | 0.429 | 2.0E-17 | 0.667 |
| MER121 | Unknown | 152 | 0.421 | 6.5E-08 | 0.754 |
| MARNA | DNA/Mariner | 121 | 0.371 | 2.7E-05 | 0.498 |
| L3b | LINE/CR1 | 395 | 0.345 | 1.4E-14 | 0.491 |
| L1M5 | LINE/L1 | 391 | 0.342 | 3.9E-12 | 0.401 |
| L3 | LINE/CR1 | 1959 | 0.324 | 2.2E-49 | 0.349 |
|  |  |  |  |  |  |

Repeat subclasses of strong rate correlation between rodent and laurasiatheria. The z-score here is defined as
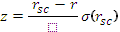
 where
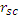
is a correlation of a repeat subclass(sc),
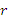
a genome wide rate correlation, and
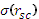
 the standard deviation among the rate correlation among all the repeat subclasses. This z-score is to qualitatively rank the strength of these correlations.
