# Supplementary material for: Weak preservation of local neutral substitution rates across mammalian genomes
Source: BMC Evol Biol. 2009 May 5;9:89. doi: 10.1186/1471-2148-9-89 (PMC2689173; doi:10.1186/1471-2148-9-89)
Supplement: Additional file 5 — Average substitution rates of the repeat subclasses 7SK, Charlie8, MER103, Charlie11, MER121, and MARNA. The table shows lower than average substitution rates of the repeat subclasses in all three lineages. [file 1471-2148-9-89-S5.doc]

Additional file 5

Sub1 is the average substitution rate in lineage 1 for the repeat family, and likewise for sub2.

%diff1 is the fractional difference in sub1 for the family with respect to the genome wide average, and likewise for %diff2. Note that all %diff values are negative for these families in both lineages in all three comparisons, strongly suggesting these families are under selection.

| Lineage1 | Lineage2 | Blocks | sub1 | sub2 | %diff1 | %diff2 |
| --- | --- | --- | --- | --- | --- | --- |
| ####7SK |  |  |  |  |  |  |
| Primate | Rodent | 14 | 0.0335 | 0.0566 | -0.4116 | -0.5569 |
| Primate | Laurasiatheria | 98 | 0.0481 | 0.1991 | -0.1562 | -0.1762 |
| Laurasiatheria | Rodent | 7 | 0.1189 | 0.0379 | -0.5081 | -0.7035 |
| ####Charlie8 |  |  |  |  |  |  |
| Primate | Rodent | 190 | 0.0486 | 0.0999 | -0.1481 | -0.2179 |
| Primate | Laurasiatheria | 1119 | 0.0522 | 0.2229 | -0.0838 | -0.0776 |
| Laurasiatheria | Rodent | 52 | 0.1759 | 0.0818 | -0.2722 | -0.3601 |
| ####MER103 |  |  |  |  |  |  |
| Primate | Rodent | 95 | 0.0526 | 0.1030 | -0.0781 | -0.1941 |
| Primate | Laurasiatheria | 2499 | 0.0502 | 0.2099 | -0.1186 | -0.1314 |
| Laurasiatheria | Rodent | 57 | 0.1945 | 0.1016 | -0.1951 | -0.2044 |
| ####Charlie11 |  |  |  |  |  |  |
| Primate | Rodent | 8 | 0.0421 | 0.1195 | -0.2612 | -0.0647 |
| Primate | Laurasiatheria | 96 | 0.0513 | 0.1917 | -0.0993 | -0.2070 |
| Laurasiatheria | Rodent | 9 | 0.1513 | 0.1157 | -0.3740 | -0.0946 |
| ####MER121 |  |  |  |  |  |  |
| Primate | Rodent | 199 | 0.0286 | 0.0593 | -0.4985 | -0.5356 |
| Primate | Laurasiatheria | 896 | 0.0313 | 0.0982 | -0.4507 | -0.5939 |
| Laurasiatheria | Rodent | 153 | 0.0890 | 0.0591 | -0.6318 | -0.5377 |
| ####MARNA |  |  |  |  |  |  |
| Primate | Rodent | 143 | 0.0387 | 0.0855 | -0.3204 | -0.3306 |
| Primate | Laurasiatheria | 1595 | 0.0459 | 0.1853 | -0.1946 | -0.2333 |
| Laurasiatheria | Rodent | 121 | 0.1559 | 0.0847 | -0.3551 | -0.3368 |
