# Supplementary material for: Weak preservation of local neutral substitution rates across mammalian genomes
Source: BMC Evol Biol. 2009 May 5;9:89. doi: 10.1186/1471-2148-9-89 (PMC2689173; doi:10.1186/1471-2148-9-89)
Supplement: Additional file 6 — CpG removed data sets. The file contains scattering plots and a table for total base pairs, the number of blocks and the average size of blocks used for each lineage. [file 1471-2148-9-89-S6.doc]

Additional file 5

r=0.103 r=0.105 r=0.223


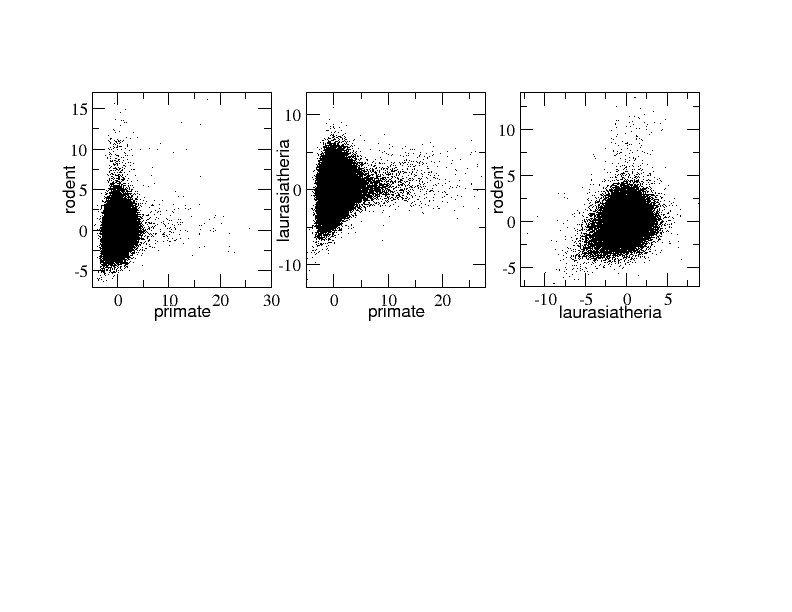


CpG removed data sets. Scatter plots of normalized substitution rates among the 3 quartets for the primate, rodent and laurasiatheria pairs.

| CpG removed | primate | rodent | primate | laurasia-theria | laurasia-theria | rodent |
| --- | --- | --- | --- | --- | --- | --- |
| Correlation | 0.103 | | 0.105 | | 0.223 | |
| p-value | 5.1E-280 | | 0 | | 0 | |
| Total block | 118813 | | 645516 | | 49502 | |
| Total (bp) | 17.50M | 11.98M | 75.44M | 61.36M | 5.98M | 5.06M |
| Average size (bp) | 147 | 100 | 116 | 96 | 120 | 102 |
