# Supplementary material for: Weak preservation of local neutral substitution rates across mammalian genomes
Source: BMC Evol Biol. 2009 May 5;9:89. doi: 10.1186/1471-2148-9-89 (PMC2689173; doi:10.1186/1471-2148-9-89)
Supplement: Additional file 7 — The analysis based on phastCons conservation score. Statistics of correlations and raw data classified based on phastCons conservation score of 17 species alignments illustrate the effects of purifying selection. [file 1471-2148-9-89-S7.doc]

Additional file 6

Statistics of correlations and raw data classified based on phastCons conservation score of 17 species alignments; a) the blocks with conservation score ≥ 0.9. b) the blocks with conservation score < 0.9.

A)

| High Conservation | primate | rodent | primate | laurasia-theria | laurasia-theria | rodent |
| --- | --- | --- | --- | --- | --- | --- |
| Correlation | 0.1133 | | 0.1730 | | 0.2473 | |
| p-value | 5.4e-37 | | 1.9e-282 | | 4.9e-92 | |
| Total block | 12476 | | 42415 | | 6561 | |
| Total (bp) | 2.4M | 1.7M | 7.3M | 6.0M | 1.06M | 0.91M |
| Average size (bp) | 188 | 134 | 174 | 142 | 162 | 138 |

B)

| Low Conservation | primate | rodent | primate | laurasia-theria | laurasia-theria | rodent |
| --- | --- | --- | --- | --- | --- | --- |
| Correlation | 0.0451 | | 0.0898 | | 0.0640 | |
| p-value | 2.7e-49 | | 0 | | 1.8e-40 | |
| Total block | 106533 | | 604278 | | 43088 | |
| Total (bp) | 15.5M | 10.6M | 69.7M | 57.0M | 5.1M | 4.3M |
| Average size (bp) | 145 | 100 | 115 | 94 | 118 | 100 |
